# The Contribution of Antibiotic Resistance Mechanisms in Clinical *Burkholderia cepacia* Complex Isolates: An Emphasis on Efflux Pump Activity

**DOI:** 10.1371/journal.pone.0104986

**Published:** 2014-08-25

**Authors:** Sung-Pin Tseng, Wan-Chi Tsai, Chih-Yuan Liang, Yin-Shiou Lin, Jun-Wei Huang, Chung-Yu Chang, Yu-Chang Tyan, Po-Liang Lu

**Affiliations:** 1 Department of Medical Laboratory Science and Biotechnology, College of Health Sciences, Kaohsiung Medical University, Kaohsiung, Taiwan, ROC; 2 Department of Laboratory Medicine, Kaohsiung Medical University Hospital, Kaohsiung, Taiwan, ROC; 3 College of Medicine, Kaohsiung Medical University, Kaohsiung, Taiwan, ROC; 4 Department of Medical Imaging and Radiological Sciences, Kaohsiung Medical University, Kaohsiung, Taiwan, ROC; 5 Department of Internal Medicine, Kaohsiung Medical University Hospital, Taiwan, ROC; University of Zurich, Switzerland

## Abstract

Due to the limited information of the contribution of various antibiotic resistance mechanisms in clinical *Burkholderia cepacia* complex isolates, Antibiotic resistance mechanisms, including integron analysis, identification of quinolone resistance-determining region mutations, measurement of efflux pump activity, and sequence analysis of efflux pump regulators, were investigated in 66 clinical *B. cepacia* complex isolates. Species were identified via *recA*-RFLP and MALDI-TOF. Four genomovars were identified by *recA*-RFLP. *B. cenocepacia* (genomovar III) was the most prevalent genomovar (90.1%). Most isolates (60/66, 90.9%) were correctly identified by MALDI-TOF analysis. Clonal relatedness determined by PFGE analysis revealed 30 pulsotypes, including two major pulsotypes that comprised 22.7% and 18.2% of the isolates, respectively. Seventeen (25.8%) isolates harboured class 1 integron with various combinations of resistance genes. Among six levofloxacin-resistant isolates, five had single-base substitutions in the *gyrA* gene and three demonstrated efflux pump activities. Among the 42 isolates exhibiting resistance to at least one antimicrobial agent, 94.4% ceftazidime-resistant isolates (17/18) and 72.7% chloramphenicol-resistant isolates (16/22) demonstrated efflux pump activity. Quantitation of efflux pump RNA level and sequence analysis revealed that over-expression of the RND-3 efflux pump was attributable to specific mutations in the RND-3 efflux pump regulator gene. In conclusion, high-level expression of efflux pumps is prevalent in *B. cepacia* complex isolates. Mutations in the RND-3 efflux pump regulator gene are the major cause of efflux pump activity, resulting in the resistance to antibiotics in clinical *B. cepacia* complex isolates.

## Introduction

The *Burkholderia cepacia* complex is comprised of Gram-negative, non-fermenting bacilli that are commonly found in natural and hospital environments [1], [2]. The *B. cepacia* complex includes at least nine species [2], [3]. Among *B. cepacia* complex infection in cases of cystic fibrosis, *B. multivorans* (genomovar II) and *B. cenocepacia* (genomovar III) are common and may cause outbreaks [1], [2]. *B. cepacia* complex has emerged as important pathogens in patients with cystic fibrosis and chronic granulomatous disease over the past two decades [4]–[6]. The difficulty in treating *B. cepacia* complex infections is attributed to its intrinsic resistance to multiple antibiotics and disinfectants [2], [7], [8]. The causes of multiple antibiotic resistance in the *B. cepacia* complex include decreased outer membrane permeability [9], [10], alterations to antibiotic targets [11], integrons [12], [13], and active efflux pumps [14]–[17]. Four RND (resistance-nodulation-division) efflux pumps, including RND-3, RND-4, RND-9 and RND-10, were shown to facilitate multiple antibiotic resistance in laboratory reference strains of the *B. cepacia* complex, whereas any contribution of these efflux pumps to resistance of clinical isolates has not been reported [14], [15], [17].

Because the contribution of various antibiotic resistance mechanisms in clinical *B. cepacia* complex isolates is not well understood, we investigated the roles of the class 1 and 2 integrons, the quinolone resistance-determining regions (QRDRs) of topoisomerase II and IV, and active efflux pumps in 66 clinical *B. cepacia* complex isolates. We also differentiated the *B. cepacia* complex to the species level by molecular typing and matrix-assisted laser desorption ionisation time-of-flight (MALDI-TOF) analysis in order to investigate the resistance mechanisms in different species.

## Materials and Methods

### Bacterial isolates

A total of 66 *B. cepacia* complex isolates were identified with the Vitek 2 system (bioMérieux) from blood (n = 45), sputum (n = 10), lung abscesses (n = 5), urine (n = 3), surgical wounds (n = 2), and pus (n = 1) between 2009 and 2011 at Kaohsiung Medical University Hospital, a 1,600-bed medical centre in Taiwan. Antibiotic resistant mechanisms of bacterial isolates were analysed in this study whereas the human specimens or patient's information were not included.

### Ethics statement

This study was approved by the Institutional Review Board of Kaohsiung Medical University Chung-Ho Memorial Hospital, Kaohsiung, Taiwan (KMUH-IRB-20130181). The study subjects were bacterial isolates and the informed consents were waived.

### Restriction fragment length polymorphism (RFLP) analyses and sequence of the *recA* gene

The genomovar typing of *B. cepacia* complex isolates was described by Mahenthiralingam *et al.* [18]. Briefly, *recA* PCR was performed with the primer pair consisting of BCR1 and BCR2 (Table 1), followed by *Hae*III digestion of the PCR products. Species were identified based on *recA*-RFLP types according to previous studies [18], [19]. After identifying five *recA*-RFLP patterns, the complete *recA* gene was sequenced in two 500-bp fragments using combinations of the PCR primers BCR1, BCR2, BCR3, and BCR4 (Table 1); nucleotide sequences were compared with sequence database using the BLAST sequence algorithm (National Center for Biotechnology Information).

**Table 1:** Primers used in this study.

Target	Primer name	Sequence (5′ to 3′)	Amplicon size (bp)	Reference
*int1*	Int1F	CGCGGCATAGACTGTAC	921	[13]
	Int1R	TTCGAATGTCGTAACCGC		
*int2*	Int2F	GCAAATGAAGTGCAACGC	465	[13]
	Int2R	ACACGCTTGCTAACGATG		
*sul1*	sul1F	CTTCGATGAGAGCCGGCGGC	437	[12]
	sul1R	GCAAGGCGGAAACCCGCGCC		
Class I integron cassette	5′CS	GGCATCCAAGCAGCAAGC	variable	[12]
	3′CS	AAGCAGACTTGACCTGAT		
Class II integron cassette	125′CS	TTTTTGTGCTGCCATATCCGTG	variable	[24]
	23′CS	TGGGCTGAGAGAGTGGT		
*gyrA*	gyrA-F	ATCTCGATTACGCGATGAGC	449	[11]
	gyrA-R	GCCGTTGATCAGCAGGTT		
*parC*	parC-F	ATTGGTCAGGGTCGTGAAGA	229	[11]
	parC-R	GTAGCGCAGCGAGAAATCCT		
*recA*	BCR1	TGACCGCCGAGAAGAGCAA	1043	[18]
	BCR2	CTCTTCTTCGTCCATCGCCTC		
*recA* sequencing primers	BCR3	GTCGCAGGCGCTGCGCAA		[18]
	BCR4	GCGCAGCGCCTGCGACAT		
BCAM0918 (control gene)	0918F	GAGATGAGCACCGATCACAC	143	[26]
	0918R	CCTTCGAGGAACGACTTCAG		
BCAL1674 (RND-3)	1674F	TTGTATCGGCGGCGAATGAT	129	This study
	1674R	CTTGTCGCCCTTTCCGCATC		
BCAL2822 (RND-4)	2822F	GCGGTGTTCCCGAACCCGAAT	168	[25]
	2822R	GCTCGACCTTGTTGCTGGCGT		
BCAM1947 (RND-9)	1947F	CGACGTCGCAGGAAGAACTC	118	This study
	1947R	ACTTCGGTGAAGCCGAGATT		
BCAM2551 (RND10)	2551F	AACACCGACCAGGACAAGAA	84	This study
	2551R	CTGCATCCCTTGCTGCACTT		
BCAL1672 (RND-3 regulator)	1672F	AGTCTACCGATGTTCCGCAAA	930	This study
	1672R	GCCACAGGGTGCGTTTGTTA		
BCAL2823 (RND-4 regulator)	2823F	GGGGTTGCGGACCCTATATT	855	This study
	2823R	AATTTCGCGGCGGTGATGTC		
BCAM1948 (RND-9 regulator)	1948F	CCGTTAAATTCGTCCGCGAG	944	This study
	1948R	ATACGCGTTTCTTCGGCGAC		
BCAM2554 (RND-10 regulator)	2554F	TAGAGACGCAGCACGATGTC	1419	This study
	2554R	GGTTTACTGCGGATTCGGGA		

### Matrix-assisted laser desorption ionisation time-of-flight (MALDI-TOF) analysis

*B. cepacia* complex isolates were grown on blood agar and incubated for 24 h at 37°C. Sample preparation and crystallisation were performed as described by Degand *et al.* [20]. MALDI-TOF MS was performed using a MALDI Biotyper system (Microflex LT, Bruker Daltonik GmbH, Bremen, Germany) and a 60-Hz nitrogen laser (337 nm wavelength). Spectra were collected in the linear positive mode in the mass range of 2,000 to 20,000 m/z for bacterial identification using MALDI Biotyper software (version 3.1.66; Bruker Daltoniks GmbH, Bremen, Germany).

### Antimicrobial susceptibility testing

Antimicrobial susceptibility testing was performed by the standard broth microdilution method according to the guidelines of the Clinical and Laboratory Standards Institute (CLSI) [21]. The MIC was defined as the lowest concentration of antibiotic that prevented bacterial growth after 20 to 24 h of incubation at 37°C. The following antimicrobial agents were tested: ceftazidime, meropenem, minocycline, trimethoprim/sulfamethoxazole, ticarcillin/clavulanic acid, levofloxacin, and chloramphenicol.

### Efflux pump activity testing

To evaluate the efflux pump activity of *B. cepacia* complex isolates, MIC resistance patterns were measured with the efflux pump inhibitor carbonyl cyanide-m-chlorophenyl hydrazone (CCCP; Sigma- Aldrich, St. Louis, MO) by the broth microdilution method [22]. Briefly, 100 µl of the final bacterial inoculation was adjusted to 5×10^5^ CFU/ml in cation-adjusted Mueller-Hinton broth (CAMHB) and incubated with 100 µl of serial two-fold dilutions of antibiotics in a 96-well plate. Subsequently, 1.2 µl CCCP (10 mM in dimethyl sulfoxide [DMSO]) was added to a final concentration of 60 µM. The MIC was defined as the lowest concentration of antibiotic that prevented bacterial growth after 20 to 24 h of incubation at 37°C. Given that 240 µM and 120 µM CCCP inhibited the growth of *B. cepacia* complex strains, a final concentration of 60 µM CCCP was used in this test. Presence of efflux pump activity was defined as any strain exhibiting at least a 4-fold decrease in MIC with CCCP.

### Pulsed-field gel electrophoresis (PFGE)

PFGE typing of DNA digested with *Xba*I (New England BioLabs, Ipswich, MA) was performed in accordance with previously described methods [23]. Restriction fragments ranging from 50 to 500 kb in size were separated on a CHEF Mapper apparatus (Bio-Rad Laboratories, Hercules, CA) for 20 h at 200 V and 14°C. The gels were then stained with ethidium bromide and photographed under UV light. Dice similarity indices were employed to construct a dendrogram of pulsotype relationships via the unweighted pair group method using arithmetic averages (UPGMA) with BioNumerics software version 6.5 (Applied Maths, Sint-Martens-Latem, Belgium). Pulsotypes were assigned to the same clusters if they exhibited 80% similarity in the dendrogram.

### PCR amplification and DNA sequencing

Genomic DNA was extracted with the Pure gene DNA Isolation Kit (Gentra Systems, Inc., Minneapolis, Minn.). PCR assays were performed to amplify the full sequences of the class 1 and 2 integron cassettes (*int*1 and *int*2) (Table 1) [12], [13], [24]. To detect quinolone resistance-determining regions (QRDRs) in topoisomerase II and IV, the *gyrA* and *parC* genes were sequenced. 4 RND efflux pump regulators (BCAL1672, BCAL2823, BCAM1948, and BCAM2554) were also sequenced. Primers used for amplification of the sequences are listed in Table 1 [11]. Purified PCR amplicons were sequenced via the dideoxy chain-termination method with an automated DNA Sequencer (Perkin-Elmer ABI3700), and nucleotide sequences were compared with sequence database using the BLAST sequence algorithm (National Center for Biotechnology Information).

### RNA extraction and synthesis of cDNA

Total RNA was extracted from 5 ml of log-phase cultures using TRIzol reagent (Life Technologies, Carlsbad, CA, USA) according to the manufacturer's recommendations. Contaminating DNA was removed with DNase I (Life Technologies, Carlsbad, CA, USA) digestion for 45 min at 37°C, followed by phenol-chloroform extractions, isopropanol precipitation, and resuspension of total RNA in nuclease-free water. Reverse transcription was performed with 5 µg RNA using random hexamers and M-MLV reverse transcriptase (Life Technologies, Carlsbad, CA, USA).

### Quantitative reverse transcription-PCR for efflux pump (efflux pump RNA expression)

PCR reactions were performed in 96-well plates in reaction buffer containing 1× FastStart Universal SYBR Green Master (Roche), 300 nM primers and 2 µl cDNA. Reactions were carried out in a ABI7000 machine following the manufacturer's protocol. Forward and reverse primers were designed using Primer-BLAST and compared to the *B. cenocepacia* J2315 genome to verify their specificity. The primers are listed in Table 1 [25], [26]. The non-induced efflux strain No. 30 was designated as a reference strain. Relative fold changes in the transcript level of indicated genes were normalised to the BCAM0918 gene and calculated with the 2^−*ΔΔCT*^ method. The BCAM0918 gene, which was stably expressed under antibiotics, was evaluated as an internal control [26].

### Statistical analysis

Categorical variables were analysed with the Chi-square test. The Mann-Whitney U test was used to determine if there were differences between isolates in terms of the expression level of efflux pumps, both for isolates that did and did not exhibit resistance to antimicrobial agents. All tests were two-tailed, and a P-value of<0.05 was considered statistically significant.

## Results

### Species identification and molecular typing

**Figure 1:**
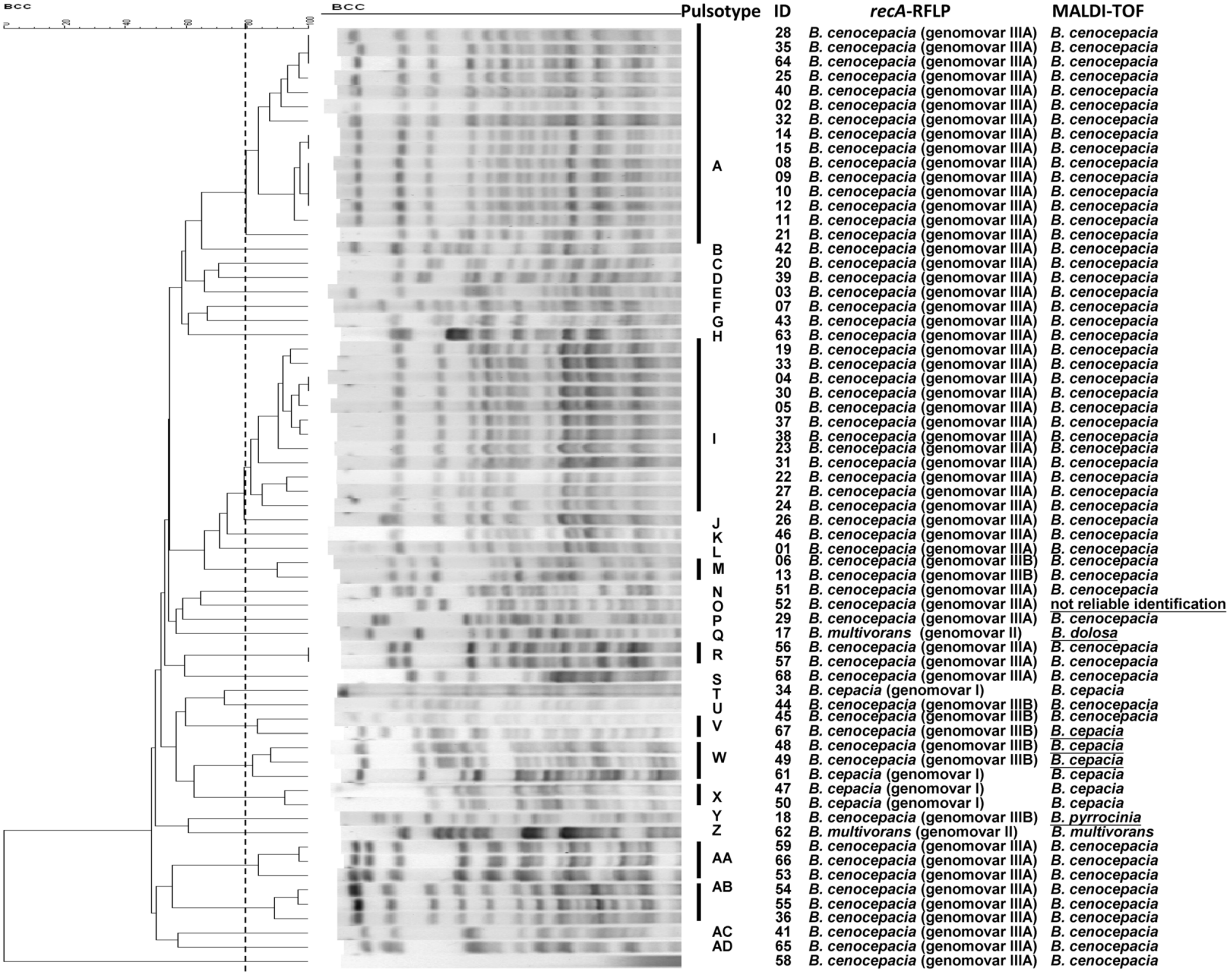
A dendrogram of pulsotype relationships developed via the unweighted pair group method using arithmetic averages (UPGMA) with BioNumerics software version 6.5 (Applied Maths). Pulsotypes were assigned to the same clusters if they exhibited 80% similarity in the dendrogram. Species identification was performed by *recA*-RFLP and MALDI-TOF analysis for 66 *B. cepacia* complex isolates.

Among 66 *B. cepacia* complex isolates, five different patterns were identified by *recA*-RFLP as the previous report [18]. Fifty-two *B. cenocepacia* (genomovar IIIA) isolates were identified as pattern G (*recA* sequence to be identical to *B. cenocepacia* J2315; accession no. AM747720). Eight *B. cenocepacia* (genomovar IIIB) isolates were identified as pattern H (*B. cenocepacia* C1394; accession no. AF143783). Four *B. cepacia* (genomovar I) isolates identified to be one isolate of pattern D (*B. cepacia* ATCC 25416; accession no. AF143786) and three isolates being pattern E (*B. cepacia* ATCC 17759; accession no. AF143788). Two *B. multivorans* (genomovar II) isolates was identified as pattern C (*B. multivorans* C1576; accession no. AF143774) (Fig. 1). Most isolates (60/66, 90.9%) were correctly identified by MALDI-TOF analysis, whereas 5 isolates (7.6%) were mistakenly identified as different species and one isolate (1.5%) had a score lower than 1.7, which indicates an unreliable identification. An isolate that was unidentified by MALDI-TOF was *B. cenocepacia* (genomovar IIIA) according to *recA*-RFLP analysis. In addition, four *B. cenocepacia* (genomovar IIIB) isolates were misidentified as *B. cepacia* (n = 3) and *B. pyrrocinia* (n = 1) by MALDI-TOF analysis. One *B. multivorans* (genomovar II) isolate was misidentified as *B. dolosa* by MALDI-TOF analysis (Fig. 1). PFGE analysis revealed 30 pulsotypes, including two major pulsotypes, A (n = 15) and I (n = 12), which were all *B. cenocepacia* (genomovar IIIA). Except for ceftazidime, no difference in the other antimicrobial resistance rates was observed between isolates of the two major pulsotypes (Table S2).

### Antimicrobial susceptibility testing

Antimicrobial susceptibility results are presented in Table 2 and the MIC data for each isolate was supplied in file S1. The susceptibility rates for minocycline and trimethoprim/sulfamethoxazole among the 66 *B. cepacia* complex isolates were highest: 90% and 97%, respectively. The susceptibility rate for meropenem was 86% and that for levofloxacin was 80%. The non-susceptibility rate for chloramphenicol was 55% and for ceftazidime was 35%. All isolates were resistant to ticarcillin/clavulanic acid. The MIC_50_ for chloramphenicol and ticarcillin/clavulanic acid were 16 (intermediate) and 128/2 mg/L (resistant), respectively. The antimicrobial susceptible rates for the tested antimicrobial agents of three species are revealed in Table S1. No significant differences in those rates between species are found.

**Table 2:** Antimicrobial susceptibilities of 66 *B. cepacia* complex isolates.

Antibiotic	MIC (µg/ml)			No. (%) of total isolates		
	Range	MIC_50_	MIC_90_	S	I	R
Chloramphenicol	4–128	16	64	30 (45)	15 (23)	21 (32)
Ceftazidime	2–128	8	64	43 (65)	7 (11)	16 (24)
Meropenem	1–128	4	16	57 (86)	0 (0)	9 (14)
Levofloxacin	0.25–64	2	4	53 (80)	7 (11)	6 (9)
Minocycline	0.5–64	2	4	60 (90)	3 (5)	3 (5)
Ticarcillin/clavulanic acid	≥128/2	128/2	≥128/2	0 (0)	0 (0)	66 (100)
Trimethoprim/sulfamethoxazole	≤0.5/9.5-4/76	≤0.5/9.5	1/19	64 (97)	0 (0)	2 (3)

### Integron cassette analysis

**Figure 2:**
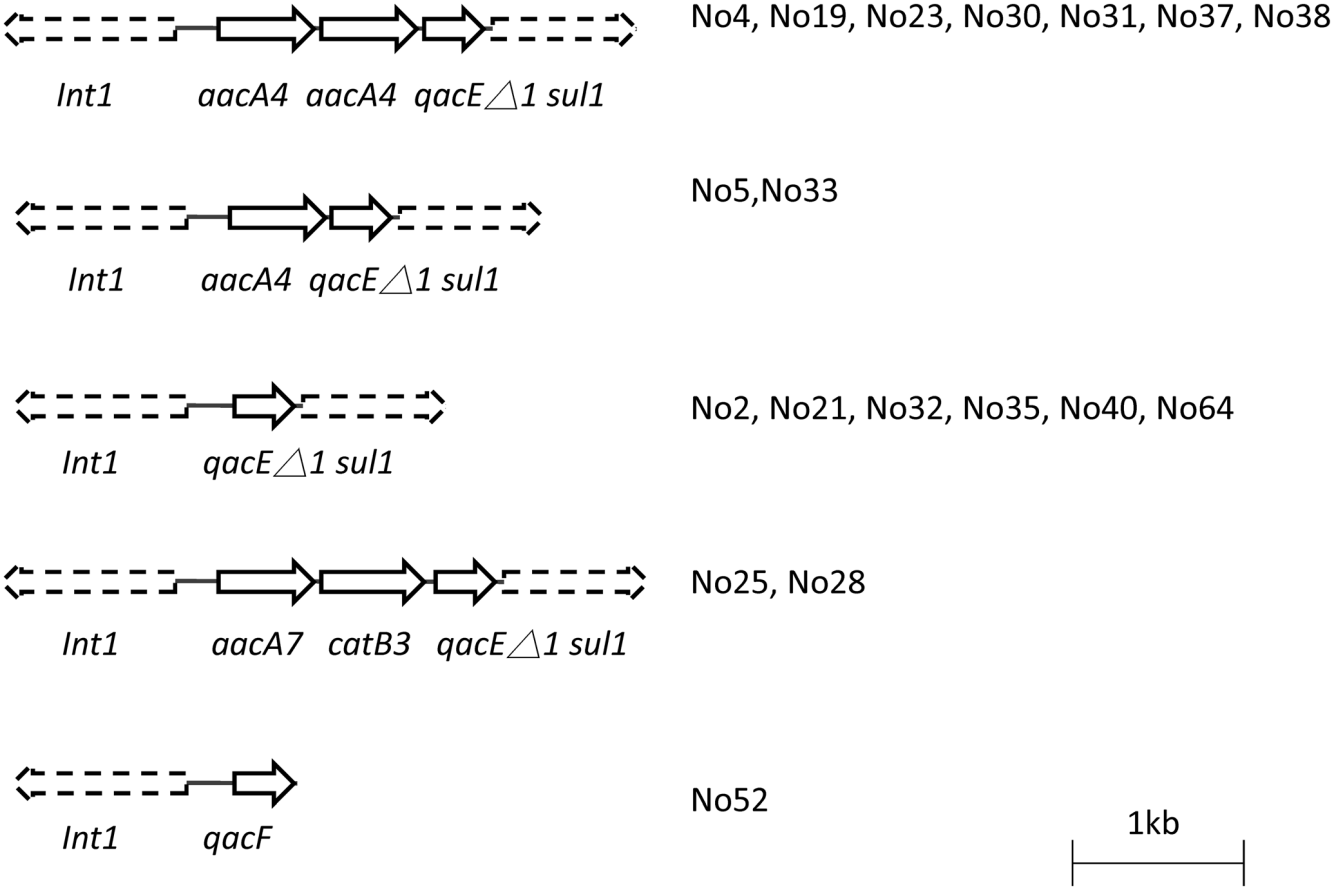
Class 1 integron cassette analysis. Genes are shown as arrows with the direction of transcription indicated by the arrowheads. *Int1*: class 1 integrase, *qacF*: quaternary ammonium compound-resistance protein, *qacEΔ1*: remnants of quaternary ammonium compound resistance protein, *sul1*: sulphonamides resistance gene, *aacA4*: aminoglycoside 6′-acetyltransferase, *aacA7*: aminoglycoside 6′-acetyltransferase, and *catB3*: chloramphenicol acetyltransferase.

Class 1 integron was identified in 18 *B. cenocepacia* isolates (Fig. 2). One isolate harboured the class 1 integrase (*intI1*) and *qacF* genes (quaternary ammonium compound-resistance protein), which were identical to the sequences for GenBank accession number FN827339. The other 17 isolates demonstrated the typical class 1 backbone structure consisting of a 5′ conserved segment (*intI1*) and a 3′ conserved segment, which included the *qacEΔ1* and sulfamethoxazole resistance gene (*sul1*). Six isolates exhibited the In0 structure (GenBank accession number: M73819.1), which is an ancestor of more complex integrons [27]. Among 11 isolates containing aminoglycoside resistant genes, 2 isolates harboured one copy of *aacA4* gene, which encodes aminoglycoside 6′-N-acetyltransferase, in the class 1 integron cassette, and 7 isolates contained two copies of the *aacA4* gene in the class 1 integron cassette (Fig. 2). *aacA7*, which encodes aminoglycoside 6′-N-acetyltransferase was found in the integrons of two isolates. Two isolates with integron containing *catB3* gene, which encodes chloramphenicol acetyltransferase were resistant to chloramphenicol. The result revealed integron's role in the resistance to sulfamethoxazole, chloramphenicol, and aminoglycoside.

### Effect of efflux pumps on antibiotic MICs (efflux pump activity)

**Figure 3:**
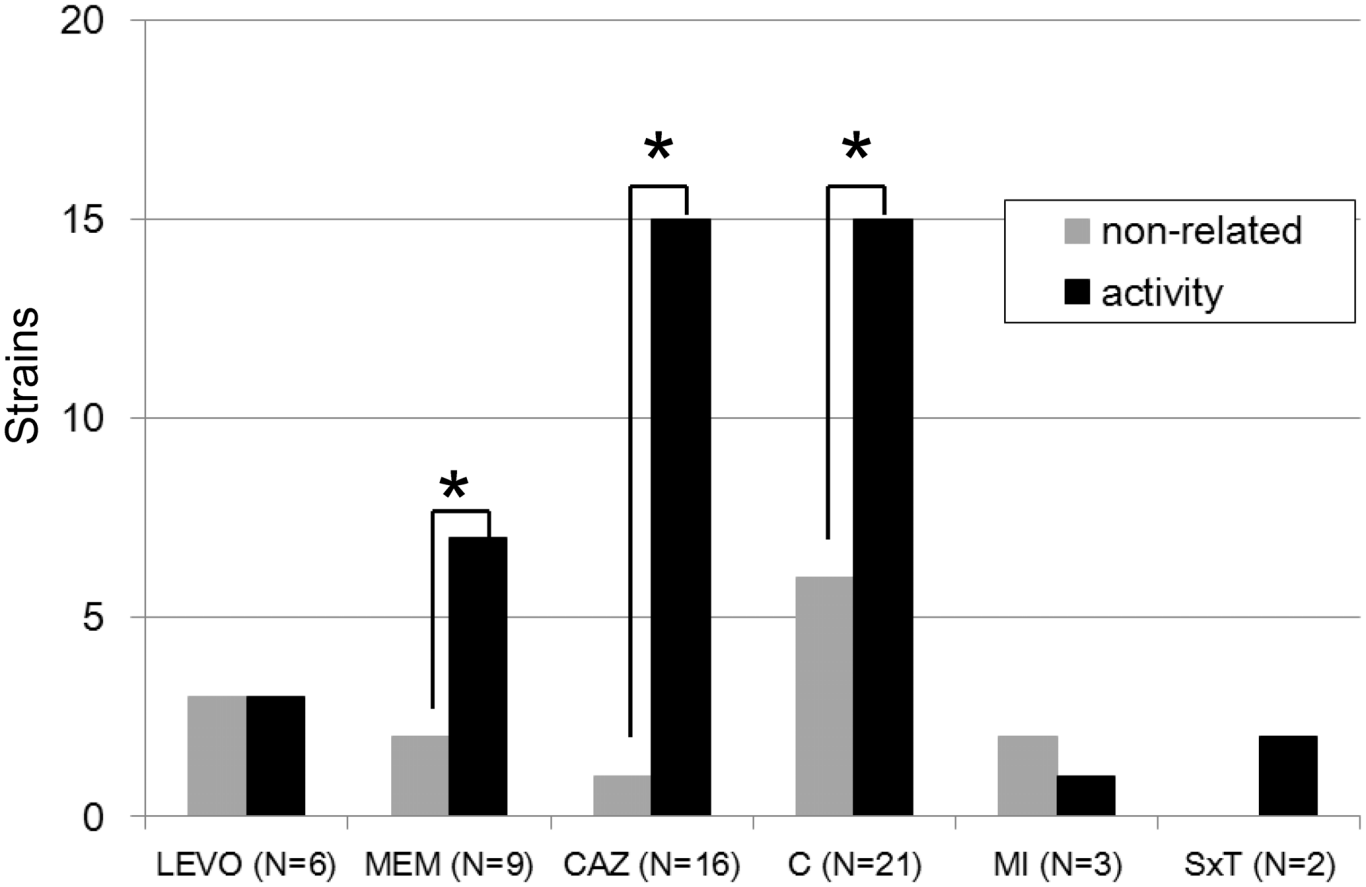
Among 42 antibiotic-resistant clinical isolates, the efficacy of efflux pumps was tested via antibiotic susceptibilities: ceftazidime (CAZ), meropenem (MEM), minocycline (MI), trimethoprim/sulfamethoxazole (SxT), levofloxacin (LEVO), and chloramphenicol (C) in the presence or absence of 60 µM CCCP. Efflux pump activity was defined as present at least 4-fold decrease of MIC in the presence of CCCP. *, *p*<0.05 (statistical analysis using the Mann-Whitney U test).

To understand the contribution of efflux pumps to antibiotic resistance, 42 isolates that were resistant to at least one antimicrobial agent, including ceftazidime, meropenem, minocycline, trimethoprim/sulfamethoxazole, levofloxacin, and chloramphenicol, were utilised for further testing. The detailed results of efflux pump activities were revealed in File S1 (CCCP method). 78.6% isolates (33/42) demonstrated presence of efflux pump activity. The proportions of strains exhibiting efflux pump activity for various antimicrobial agents are revealed in Fig. 3. 50% of levofloxacin-resistant isolates (3/6) and 70% meropenem-resistant isolates (7/10) demonstrated efflux pump activity. Most ceftazidime-resistant (17/18, 94.4%) and chloramphenicol-resistant (16/22, 72.7%) isolates also exhibited efflux pump activity. Besides, efflux pump activity was identified in both trimethoprim/sulfamethoxazole-resistant isolates in the study. The presence of efflux pump activity was significantly correlated with resistance to any of the following antimicrobial agents: ceftazidime, meropenem, and chloramphenicol (all *p*<0.05 according to the Mann-Whitney U test).

### Characteristics of levofloxacin-resistant mechanisms

Resistant mechanism of levofloxacin has been known via accumulation of mutations in the QRDR of topoisomerase genes and efflux pump activation [7], [11]. Among the 66 *B. cepacia* complex isolates, resistance to levofloxacin was identified in 6 clinical isolates. Sequence analysis determined that single-base substitutions in the QRDR regions of the *gyrA* gene occurred at codons 81 (Gly81Asp), 83 (Thr83Ile), and 87 (Asp87His). No mutation was found in the QRDR region of the *parC* gene. Besides, higher efflux pump activity was found in 50% levofloxacin- resistant isolates (3/6) and could be related to levofloxacin resistance (Table 3).

**Table 3:** Characteristics of QRDR mutations and efflux pump activity of six fluoroquinolone-resistant *B. cepacia* complex isolates.

Strain	MIC of levofloxacin (µg/ml)		Efflux pump activity	Mutation in	
	CCCP (−) CCCP (+)			*gyrA* gene	*parC* gene
No. 1	8	8	none	Thr83Ile (ACC→ATC)	no amino acid change
No. 21	8	2	activity	no amino acid change	no amino acid change
No. 26	8	8	none	Thr83Ile (ACC→ATC)	no amino acid change
No. 27	32	8	activity	Asp87His ( GAC→ CAC)	no amino acid change
No. 34	16	16	none	Thr83Ile (ACC→ATC)	no amino acid change
No. 52	64	8	activity	Gly81Asp (GGC→GAC)	no amino acid change

### Quantitative RNA expression of RND efflux pumps (efflux pump RNA expression)

According to previous studies, four active RND pumps (RND-3, RND-4, RND-9 and RND-10) were linked to bacterial antibiotic resistance in *B. cenocepacia* laboratory stains; however, their contributions in clinical isolates had not been elucidated [14], [15], [17]. To understand whether these efflux pumps were expressed in clinical isolates, some representative isolates with efflux pump activity (no. 4, no. 23, and no. 27) and without efflux pump activity (no. 30, and no. 38) were selected from pulsotype (pulsotype I) and the transcript abundance of these four efflux pumps quantified by qRT-PCR (Table 4). Expression of RND-4 (BCAL2822) and RND-10 (BCAM2551) were no significant difference in these clinical isolates. High expression level of RND-3 (BCAL1674) and RND-9 (BCAM1947) were identified in all clinical isolates demonstrating efflux pump activity (Table 4).

**Table 4:** Quantitative RNA expression of four RND efflux systems in five selected *B. cenocepacia* (genomovar IIIA) isolates.

Efflux pump activity	Fold change in expression^a^			
	BCAL1674 (RND-3)	BCAL2822 (RND-4)	BCAM1947 (RND-9)	BCAM2551 (RND-10)
Activity				
No. 4	7.41±0.77	1.44±0.65	3.77±0.88	2.02±1.25
No. 23	6.94±0.92	1.47±0.89	2.99±0.54	1.78±0.56
No. 27	8.54±1.31	1.00±0.27	3.47±0.73	0.88±0.53
Non-induced				
No. 30	1^b^	1^b^	1^b^	1^b^
No. 38	0.87±0.05	1.09±0.22	0.74±0.17	1.34±0.11

a . Fold change was determined by quantitative reverse transcription-PCR (quantitative RT-PCR).

b . No. 30 was a strain with a non-induced efflux pump that was utilised as a reference strain.

### Sequence analysis of regulators in RND efflux pump genes

**Figure 4:**
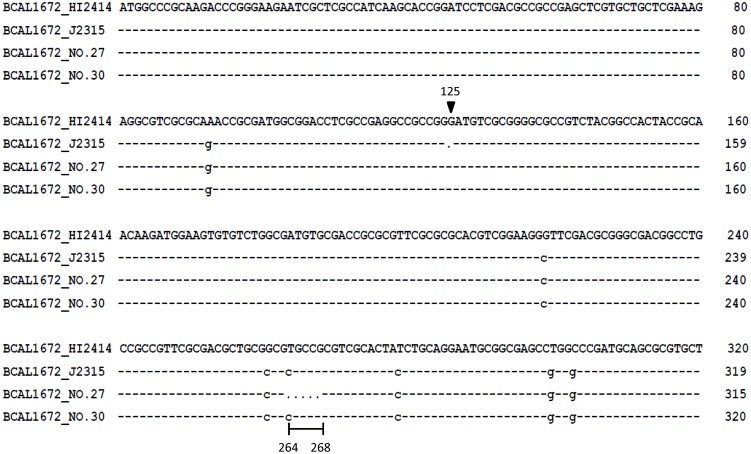
Comparison of the BCAL1672 gene (the first 320 bp) in *B. cenocepacia* isolates. The arrow (at position 125) indicates the deletion of a guanine nucleotide in *B. cenocepacia* J2315. Deleted nucleotides (between positions 264 and 268) were identified in clinical isolate No. 27.

Based on previous studies that determined that the over-expression of multidrug resistance efflux pumps was usually correlated with mutations in regulator genes in clinical *Pseudomonas aeruginosa* isolates [28], [29], two regulator genes (RND-3, and RND-9) including their promoter regions were sequenced in the five *B. cenocepacia* (genomovar IIIA) isolates. The regulators of RND-9 demonstrated no amino acid changes when compared to *B. cenocepacia* J2315 (GenBank accession numbers AM747720.1). Because the regulator of RND-3 (BCAL1672) is a pseudogene in *B. cenocepacia* J2315, sequence analysis of the BCAL1672 gene was compared with *B. cenocepacia* HI2424 (GenBank accession numbers CP000458.1) [30]. For three of isolates with efflux pump activity (no. 4, no. 23 and no. 27), five nucleotide deletions (from position 264 to 268) were identified in the BCAL1672 gene (GenBank accession numbers KJ882899). The deletion of a guanine nucleotide at position 125 could result in the translational frameshifting in *B. cenocepacia* J2315 (Fig. 4). The five nucleotide deletions from position 264 to 268 caused protein frameshifting. These results indicated that mutations in the BCAL1672 repressor could affect RND-3 efflux pump expression in *B. cenocepacia* clinical isolates.

### Correlation of BCAL1672 gene mutations with efflux pump expression in clinical *B. cenocepacia* (genomovar IIIA) isolates

To investigate the BCAL1672 gene, which is a regulator of the RND-3 efflux pump in clinical *B. cenocepacia* (genomovar IIIA) isolates, 40 isolates were included for analysis. Ten isolates were susceptible to all tested antimicrobials. Thirty isolates were resistant to at least one antimicrobial agnet (25 isolates with efflux pump activity and five isolates without the activity). The five nucleotide deletions constituted predominant mutations observed in 8 of 25 isolates with efflux pump activity (Table 5). One isolate had 12 nucleotide deletions at the end of BCAL1672 (from position 586 to 598), and six isolates harboured a Ser174Pro (TCG→CCG) variation. When the groups demonstrating efflux pump activity and without activity were compared, these three variations of BCAL1672 were statistically correlated with RND-3 efflux pump activity (Chi-square test, *p*<0.05). Investigation of these three mutants and antibiotic resistance in these 25 isolates with efflux pump activity found that mutation of BCAL1672 could be related to chloramphenicol (12/14; 86%), meropenem (3/5; 60%), and levofloxacin (2/3; 67%) resistance. However, only three ceftazidime resistant isolates had such mutations (3/10; 30%) (Table 6). These results indicated that mutations of the BCAL1672 gene are associated with efflux pumps activity causing antibiotic resistance whereas ceftazidime resistance could be related to other unidentified mechanisms.

**Table 5:** Sequence analysis of regulators for the RND-3 efflux systems in *B. cenocepacia* (genomovar IIIA).

Mutation in BCAL1672 (RND-3 regulator)^a^	Efflux activity isolates (N = 25)	Non-induced (N = 5) and all susceptible isolates (N = 10)^b^
**264 GCGTG 268; 5-nt deletion** c	8	0
**586 GCTTCGACGCCG 598; 12 nt deletion** c	1	0
Ala140Val (GCG→GTG)	1	0
Val153Ala (GTC→GCC)	16	15
**Ser174Pro ( TCG→ CCG)** c	6	0
Ala199Thr ( GCC→ ACC)	1	6
Arg208Gly ( CGA→ GGA)	16	6
Arg208Gly ( CGA → GGG )	0	9
Asp215Ser ( GAC→ AGC)	1	0

a . Sequence compared with *B. cenocepacia* HI2424 (GenBank accession number: CP000458.1).

b . All susceptible isolates were tested for their antimicrobial susceptibilities to CAZ, LVX, C, MEM, MI, and SXT.

c . The variations in BCAL1672 that are related to RND-3 efflux pump activity are indicated in bold.

**Table 6:** Correlation between BCAL1672 mutants and antibiotic resistance in 25 efflux activity *B. cenocepacia* (genomovar IIIA) isolates.

Mutation in BCAL1672 (RND-3 regulator)^a^	Antibiotic resistance^b^					
	C (n = 14)	CAZ (n = 10)	MEM (n = 5)	LVX (n = 3)	MI (n = 1)	SXT (n = 2)
**264 GCGTG 268; 5-nt deletion** c (n = 8)	8	1	0	0	0	2
**586 GCTTCGACGCCG 598; 12 nt deletion** c (n = 1)	1	0	1	1	0	0
**Ser174Pro ( TCG→ CCG)** c (n = 6)	3	2	2	1	0	0

a . Sequence compared with *B. cenocepacia* HI2424 (GenBank accession number: CP000458.1).

b . C = chloramphenicol, CAZ = ceftazidime, MEM = meropenem, LVX = levofloxacin, MI = minocycline, and SXT = Trimethoprim/sulfamethoxazole

c . The variations in BCAL1672 that are related to RND-3 efflux pump activity are indicated in bold.

## Discussion

Most studies of the *B. cepacia* complex in Taiwan were reported at least ten years ago [31]–[33]. Here, we present the updated data revealing that the non-susceptibility rate for ceftazidime increased from 13.9% (a previous report in 2011) [5] to 35% (this study). All 66 isolates (100%) studies were resistant to ticarcillin/clavulanic acid and the non-susceptibility rates of chloramphenicol and ceftazidime were 55% and 35%, respectively (Table 2). The above indicated antimicrobial resistance of *B. cepacia* complex to cause a serious clinical concern. Seventeen of 18 ceftazidime-resistant isolates and 16 of 22 chloramphenicol-resistant isolates exhibited efflux pump activity (Fig. 3). Half of the levofloxacin-resistant isolates demonstrated efflux pump activity resulting in at least a 4-fold change in MIC. These data indicate that efflux pump activity plays an important role in causing antibiotic resistance in the *B. cepacia* complex.

In recent decades, efflux pump activity has emerged and become a significant resistant mechanism in many species of bacteria [7], [34]. *In silico* analysis of the *B. cenocepacia* genome sequence revealed 16 putative RND efflux pumps [35], [36]. Genetic analysis indicated that 4 active RND pumps (RND-3, RND-4, RND-9 and RND-10) were correlated with antibiotic resistance in the *B. cepacia* complex. However, the function of the RND pumps in clinical isolates was not clearly understood [14], [15], [17]. The genes associated with the RND-3 efflux pump include a regulator gene (BCAL1672) and tripartite component genes (BCAL1674 through BCAL1676). Two studies revealed that the RND-3 efflux pump was strongly induced by chloramphenicol and correlated with the extrusion of nalidixic acid [15], [35]. Our results revealed that the RND-3 efflux pump is the most up-regulated among the RND pumps tested in clinical isolates. Quantitative RT-PCR demonstrated that mutations in BCAL1672 result in the activation of the RND-3 efflux pump. Correlation of antibiotic resistance and efflux pump activation revealed that all 14 *B. cenocepacia* (genomovar IIIA) isolates with efflux pump activity were resistant to chloramphenicol. Among them, 12 had mutations in the regulator gene BCAL1672 (Table 6). The RND-9 efflux operon (comprising BCAM1945 through BCAM1947) belongs to the HAE-1 family, which includes proteins responsible for the extrusion of aminoglycosides, ethidium bromide, fluoroquinolones and β-lactams [14], [37]. In this study, three clinical isolates exhibited increased transcriptional expression of RND-9 (BCAM1947), whereas no mutations were found in the regulator BCAM1948 or the promoter region (Table 4). Up-regulation of RND-9 efflux pump expression could be activated by an unknown transcriptional factor like the situation that the global activator MarA of *E.coli* that can increase the *acrAB* efflux pump expression [38]. The efflux pump mechanisms for cefazidime resistance in *B. cenocepacia* (genomovar IIIA) isolates warrant further investigation (Table 6). Among the 6 fluoroquinolone-resistant *B. cepacia* complex isolates, amino acid changes were found in the QRDR region of the *gyrA* gene, including Gly81Asp, Thr83Ile, and Asp87His. No mutation was found in the QRDR region of the *parC* gene (Table 3). A previous study showed that fluoroquinolone resistance was correlated to a Thr83Ile or Asp87Asn mutation in the *gyrA* gene [11]. The Gly81Asp and Asp87His mutations in the *gyrA* gene in *B. cepacia* complex isolates are newly reported in this study.

Only a few studies have described integrons in the *B. cepacia* complex. Ramírez et al. described a class 2 integron that harboured a streptothricin acetyltransferase in *B. cenocepacia* (IIIB) [13]. Crowley et al. reported a class 1 integron containing *bla*_OXA_ (β-lactamase) and *aac*(6′)-1a (encoded aminoglycoside 6′-N-acetyltransferase type 1b) in *B. cenocepacia* (III) [12]. We reported class 1 integron harbouring genes in 18 isolates (18/66, 27.3%). Only one isolate contained a class 1 integrase and the *qacF* gene (quaternary ammonium compound-resistance protein); the other isolates harboured the typical integron structure (In0) and acquired different copy of the *aacA4* gene. No class 2 integron was found. Our results indicate that the class 1 integron cassette is an important vehicle to transmit resistance to aminoglycoside, sulfamethoxazole and chloramphenicol in *B. cepacia* complex isolates.

The *rec*A-RFLP typing performed that *B. cenocepacia* (IIIA and IIIB had 52 and 8 isolates, respectively) was prevalent among *B. cepacia* complex isolates. Because molecular methods consume time and are costly, the more rapid and cost efficient MALDI-TOF analysis has been evaluated for species identification. Species identification accuracy was 90.9% (60/66) for MALDI-TOF analysis. This result indicated that MALDI-TOF has the potential for application in the clinical microbiology laboratory to differentiate *B. cepacia* complex to species level. Though two major pulsotypes were identified, PFGE analysis revealed high discrimination power and high genotypic diversity of *B. cepacia* complex.

In summary, antibiotic resistance mechanisms, including mutation of the QRDR region of topoisomerase II, the presence of the class 1 integron, and overexpression of efflux pumps, were common in *B. cepacia* complex clinical isolates. Mutation of the regulator gene of the RND-3 efflux pump was a major cause of high-level efflux pump expression in clinical *B. cepacia* complex isolates. Given the contribution of efflux pumps to antibiotic resistance in clinical *B. cepacia* complex isolates, the development of antimicrobial drugs targeting efflux pumps may counteract antimicrobial-resistant bacteria in the future.

## Supporting Information

Table S1 **Antimicrobial susceptibility patterns of species of 66** ***B. cepacia*** **complex isolates.**
(DOCX)

Table S2 **Antimicrobial susceptibility patterns of pulsotype A (n = 15) and I (n = 12)** ***B. cepacia*** **complex isolates.**
(DOCX)

File S1 **Antimicrobial susceptibilities of each** ***B. cepacia*** **complex isolates, efflux pump activity testing and correlation between BCAL1672 mutants and antibiotic resistance in 25 efflux activity** ***B. cenocepacia*** **(genomovar IIIA) isolates.**
(XLSX)
