# Supplementary material for: The Contribution of Antibiotic Resistance Mechanisms in Clinical Burkholderia cepacia Complex Isolates: An Emphasis on Efflux Pump Activity
Source: PLoS One. 2014 Aug 25;9(8):e104986. doi: 10.1371/journal.pone.0104986 (PMC4143217; doi:10.1371/journal.pone.0104986)
Supplement: Table S1 — Antimicrobial susceptibility patterns of species of 66 B. cepacia complex isolates. (DOCX) [file pone.0104986.s001.docx]

**Table S1.** Antimicrobial susceptibility patterns of species of 66 *B. cepacia* complex isolates

| Antibiotic ^a^ | No. (%) of total isolates  (n = 66) | | | No. (%) of 4 *B. cepacia* isolates (genomovar I) | | | No. (%) of 2 *B. multivorans* isolates (genomovar II) | | | No. (%) of 60 *B. cenocepacia* isolates (genomovar IIIA & IIIB) | | |
| --- | --- | --- | --- | --- | --- | --- | --- | --- | --- | --- | --- | --- |
|  | S | I | R | S | I | R | S | I | R | S | I | R |
| C | 30 (45) | 15 (23) | 21 (32) | 2 (50) | 2 (50) | 0 (0) | 1 (50) | 0 (0) | 1 (50) | 27 (45) | 13 (22) | 20 (33) |
| CAZ | 43 (65) | 7 (11) | 16 (24) | 4 (100) | 0 (0) | 0 (0) | 2 (100) | 0 (0) | 0 (0) | 37 (61) | 7 (12) | 16 (27) |
| MEM | 57 (86) | 0 (0) | 9 (14) | 3 (75) | 0 (0) | 1 (25) | 1 (50) | 0 (0) | 1 (50) | 53 (88) | 0 (0) | 7 (12) |
| LVX | 53 (80) | 7 (11) | 6 (9) | 3 (75) | 0 (0) | 1 (25) | 1 (50) | 1 (50) | 0 (0) | 49 (82) | 6 (10) | 5 (8) |
| MI | 60 (90) | 3 (5) | 3 (5) | 4 (100) | 0 (0) | 0 (0) | 2 (100) | 0 (0) | 0 (0) | 54 (90) | 3 (5) | 3 (5) |
| TIM | 0 (0) | 0 (0) | 68 (100) | 0 (0) | 0 (0) | 4 (100) | 0 (0) | 0 (0) | 2 (100) | 0 (0) | 0 (0) | 60 (100) |
| SXT | 64 (97) | 0 (0) | 2 (3) | 4 (100) | 0 (0) | 0 (0) | 2 (100) | 0 (0) | 0 (0) | 58 (97) | 0 (0) | 2 (3) |

a. C= Chloramphenicol, CAZ= Ceftazidime, LVX= Levofloxacin, MEM= Meropenem, MI=Minocycline, TIM=Ticarcillin/clavulanic acid and SXT= Trimethoprim/sulfamethoxazole
