# Supplementary material for: The Contribution of Antibiotic Resistance Mechanisms in Clinical Burkholderia cepacia Complex Isolates: An Emphasis on Efflux Pump Activity
Source: PLoS One. 2014 Aug 25;9(8):e104986. doi: 10.1371/journal.pone.0104986 (PMC4143217; doi:10.1371/journal.pone.0104986)
Supplement: Table S2 — Antimicrobial susceptibility patterns of pulsotype A (n = 15) and I (n = 12) B. cepacia complex isolates. (DOCX) [file pone.0104986.s002.docx]

**Table S2.** Antimicrobial susceptibility patterns of pulsotype A (n=15) and I (n=12) *B. cepacia* complex isolates

| Antibiotic ^a^ | Pulsotype A (n=15) | | | Pulsotype I (n=12) | | |
| --- | --- | --- | --- | --- | --- | --- |
|  | S | I | R | S | I | R |
| C | 5 (33) | 7 (47) | 3 (20) | 3 (25) | 0 (0) | 9 (75) |
| CAZ | 8 (53) | 3 (20) | 4 (27) | 11 (92) | 0 (0) | 1 (8) |
| MEM | 13 (87) | 0 (0) | 2 (13) | 12 (100) | 0 (0) | 0 (0) |
| LVX | 14 (93) | 0 (0) | 1 (7) | 9 (75) | 2 (17) | 1 (8) |
| MI | 15 (100) | 0 (0) | 0 (0) | 12 (100) | 0 (0) | 0 (0) |
| TIM | 0 (0) | 0 (0) | 15 (100) | 0 (0) | 0 (0) | 12 (100) |
| SXT | 15 (100) | 0 (0) | 0 (0) | 10 (83) | 0 (0) | 2 (17) |

a. C= Chloramphenicol, CAZ= Ceftazidime, LVX= Levofloxacin, MEM= Meropenem, MI=Minocycline, TIM=Ticarcillin/clavulanic acid and SXT= Trimethoprim/sulfamethoxazole
